# Ancestral sequence reconstruction as a tool to study the evolution of wood decaying fungi

**DOI:** 10.3389/ffunb.2022.1003489

**Published:** 2022-10-14

**Authors:** Iván Ayuso-Fernández, Gonzalo Molpeceres, Susana Camarero, Francisco Javier Ruiz-Dueñas, Angel T. Martínez

**Affiliations:** ^1^ Faculty of Chemistry, Biotechnology and Food Science, Norwegian University of Life Sciences (NMBU), Ås, Norway; ^2^ Centro de Investigaciones Biológicas “Margarita Salas” (CIB), CSIC, Madrid, Spain

**Keywords:** ancestral sequence reconstruction, wood decay fungi, lignocellulosic biomass, plant cell-wall degrading enzymes (PCWDE), evolution

## Abstract

The study of evolution is limited by the techniques available to do so. Aside from the use of the fossil record, molecular phylogenetics can provide a detailed characterization of evolutionary histories using genes, genomes and proteins. However, these tools provide scarce biochemical information of the organisms and systems of interest and are therefore very limited when they come to explain protein evolution. In the past decade, this limitation has been overcome by the development of ancestral sequence reconstruction (ASR) methods. ASR allows the subsequent resurrection in the laboratory of inferred proteins from now extinct organisms, becoming an outstanding tool to study enzyme evolution. Here we review the recent advances in ASR methods and their application to study fungal evolution, with special focus on wood-decay fungi as essential organisms in the global carbon cycling.

## 1 Introduction

### 1.1 Molecular paleogenetics concept

**Figure 1:**
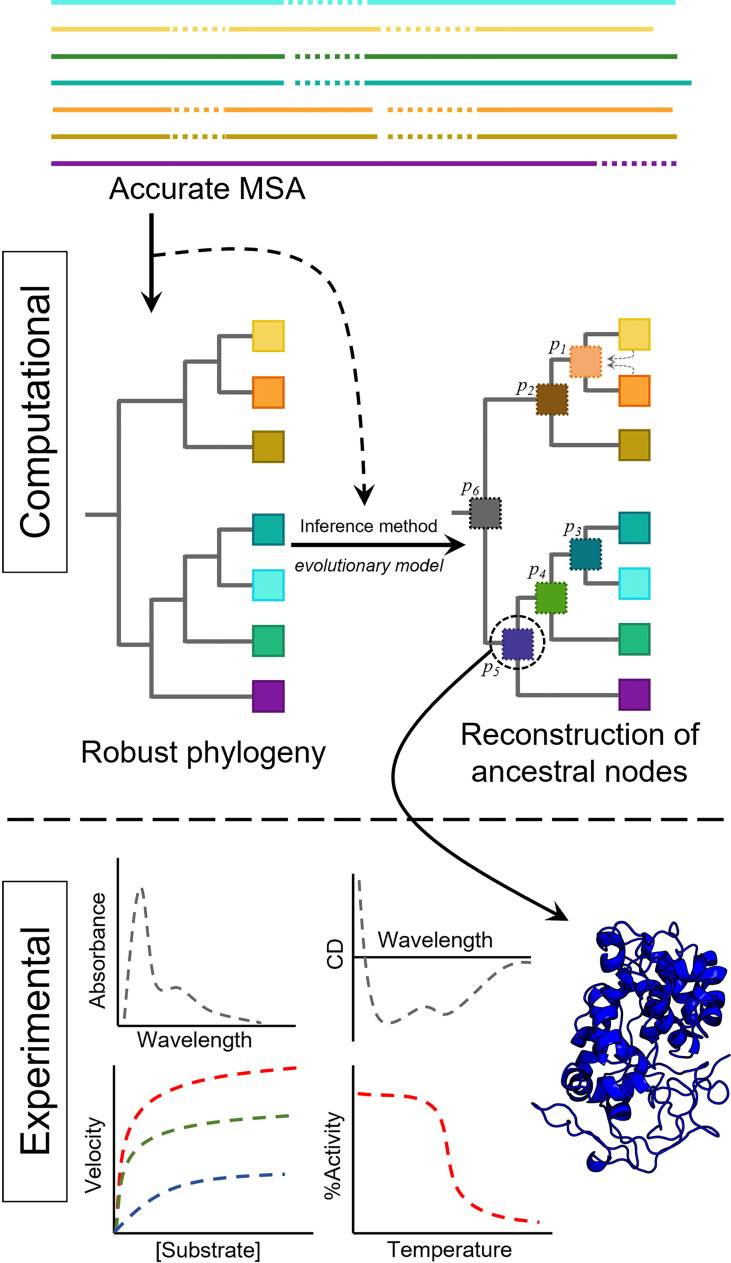
Simplified pipeline to resurrect ancestral proteins, including computational and experimental work. The sequences of the family of interest are aligned using accurate algorithms. The phylogeny is then obtained, and together with an evolutionary model and the multiple sequence alignment (MSA), the ancestral sequences at each node can be obtained. Once the ancestral sequence is selected, it can be resurrected (produced) in the laboratory to obtain the experimental data of interest (e.g. UV-vis or CD spectral properties, enzyme activity or thermal stability).

Ancestral protein resurrection opens fascinating ways to test evolutionary hypotheses otherwise impossible to be addressed. The concept of molecular resurrection stems from the seminal work by Linus Pauling and Emile Zuckerkandl, where they coined the idea of molecular paleogenetics – or how to mathematically infer the ancestral sequences of existing proteins and genes (Pauling et al., 1963). In their work, they proposed a method based on comparing existing sequences to calculate the probability of amino acids in ancestral nodes from protein phylogenies (**Figure 1**). In this way, the most probable sequence at any ancestral node of a phylogeny could be obtained, and even resurrected in the laboratory to evaluate its biochemical properties. Unfortunately, sequence information in their time was scarce, and it was not until the 90s, with the accumulation of gene (and genome) sequencing information in databases, that the first examples of ancestral proteins resurrected in the laboratory appeared (Malcolm et al., 1990; Stackhouse et al., 1990). From then on, the ideas of Pauling and Zuckerkandl were brought to life, and the field of paleogenetics started a beautiful and fascinating journey. There has been a continuous development and refinement of algorithms (and their implementation as software), together with the identification of the main caveats of ASR and ancestral protein characterization efforts in many protein families. The theoretical and experimental aspects of ASR continue to develop in parallel, and the detailed history behind ASR can be found in dedicated reviews and books (Chang and Donoghue, 2000; Liberles, 2007; Merkl and Sterner, 2016).

Today the examples of resurrected proteins cover dozens of protein families and range all fields of life sciences (Gumulya and Gillam, 2016). Some studies aim to describe the origin of life, pointing to hot ancestral environments as the broth where the first organisms appeared (Gaucher et al., 2003). Physiology and behavior of extinct species can also benefit greatly from ASR studies, as shown with the reconstruction of dinosaur vision (Chang et al., 2002) and the origins of alcohol metabolism in hominids (Carrigan et al., 2015). Analyzing the evolution of enzyme families is appealing, and outstanding works have shown how specific point mutations can restrict substrate specificity (Voordeckers et al., 2012), how plants diversified the production of secondary metabolites (Huang et al., 2012) or even how cancer-related kinases acquired resistance to drugs (Wilson et al., 2015).

But ASR is not only used in evolutionary biochemistry, it also has clear applications in industry and medicine. In this sense, outstanding examples show how ASR has been used to optimize vaccines using computational approaches, such as those targeting HIV by calculation of centralized genes that minimize the genetic distance to circulating strains (Nickle et al., 2003; Arenas and Posada, 2010b). ASR has also been proposed as a central tool for protein engineers (Spence et al., 2021), and could be used together with other tools (e.g. molecular directed evolution) to optimize industrial processes such as those related to the enzymatic degradation and valorization of biomass in the biorefinery context (Alcalde, 2015). Of course, the above is a small selection of a continuously growing number of works where ASR methods are used to study protein evolution or to take advantage of ancestral gene properties.

In this review we will focus on fungi, which stand out as one of the most diverse kingdoms of life. Among them, we will analyze how ASR has been useful to study the wood-decay fungal enzymes as essential components in the global carbon cycle. Wood-decay fungi secrete a highly diverse enzyme consortium to attack the main components of the plant cell wall, being essentially lignin, cellulose and hemicelluloses. Analyzing how they evolved and how their plant cell wall degrading enzymes (PCWDE) acquired the properties they have today is important from a fundamental and an applied point of view.

### 1.2 ASR methodology

Before diving into fungal evolution and how ASR can help its study, we will briefly describe the methodology to reconstruct ancestral sequences. Since the ultimate goal of ASR is getting proteins resurrected in the laboratory to evaluate an evolutionary hypothesis or characterize interesting properties, an ancestral resurrection experiment is only as good as the inferred sequences. Therefore, special care must be taken to obtain ancestral sequences, and a good understanding of the methods and the sources of uncertainty and ambiguity will improve the overall quality of the reconstruction. **Figure 1** shows the simplified pipeline required for enzyme resurrection. Typically, a number of protein sequences up to a few hundred are aligned, and a phylogeny is built. The phylogeny, the aligned extant sequences, and an evolutionary model describing the probabilities of change from one amino acid to another are then used to infer the ancestral sequences. Once these sequences are manually curated, the genes will be synthesized and the proteins resurrected in the laboratory, measuring the properties of interest to assess their modification through evolution.

The inference of ancestral sequences can be done with three different approaches: maximum parsimony (MP), maximum likelihood (ML) or Bayesian (often called hierarchical Bayesian, HB) methods, the two latter being the so-called probabilistic methods (Selberg et al., 2021). All three require a multiple sequence alignment (MSA) of the proteins of interest and a phylogenetic tree explaining relationships between the orthologues selected, and ML and HB methods allow the use of different models of evolution (often called substitution models) (Arenas, 2015). Taking this into account, when doing ASR, it is crucial to obtain accurate MSAs and phylogenetic trees independent of the method used to infer the ancestral sequences.

Obtaining an accurate MSA of the proteins of interest is far from trivial. The high number of algorithms available make this a difficult choice. It is known that the alignment method has an impact in the ancestral reconstruction and there is potential to introduce biases in the inferred sequences (Vialle et al., 2018), with some methods [MAFFT (Katoh et al., 2002), PRANK (Löytynoja, 2014)] performing better than others with simulated data sets. Trying to systematically analyze how important this choice is, Aadland and Kolaczkowski (2020) tested the accuracy of some of the most popular tools for sequence alignment. Their study shows that to improve accuracy, the best choice is to integrate the results of different algorithms combining the information from different sequence alignments, diminishing this way an important source for errors in the ancestral reconstruction.

The selection of protein substitution models (models of evolution) is a common practice in phylogenetic reconstruction and ASR, often done by obtaining the best-fitting model scored using likelihood-based methods (Darriba et al., 2011). However, there is debate in the field since it has been said that model selection has little importance in the final reconstructions (Abadi et al., 2019; Tao et al., 2020). To evaluate the impact of model selection in ASR, computational studies using real and simulated data have recently shown that the best-fitting model yields the most accurate sequences, and if the best-fitting model cannot be applied, the most similar models are preferred aiming to obtain the best reconstructions possible (Del Amparo and Arenas, 2022).

The phylogeny for ASR is mainly obtained with ML or Bayesian methods. The true phylogeny of the family of interest is rarely known, but some studies have shown that its uncertainty has little impact in the reconstruction (Hanson-Smith et al., 2010). In this sense, ML methods for ASR ignore the uncertainty in the phylogeny, while Bayesian methods incorporate it by sampling the distribution of ancestral states. However, using simulated data, Hanson-Smith et al. (2010) showed that Bayesian approaches to integrate over different topologies of the phylogenetic tree do not improve the accuracy of the reconstruction over ML methods. This proves that ASR is robust to uncertainty since the conditions that generate such uncertainty are also making the ancestral state identical between different trees.

Once an accurate MSA and a phylogeny are obtained, ASR can be performed with three different approaches as described above. MP methods infer the ancestral sequence that explains the minimum number of changes leading to extant proteins. Since they were the first methods employed to obtain ancestral states (Fitch, 1971), they are the less sophisticated and barely used today. ML and HB methods have been continuously improved since the early applications of ASR, and a detailed description of them can be found in other reviews (Merkl and Sterner, 2016; Selberg et al., 2021). Among the advantages of Bayesian methods, it has been argued that they integrate over the uncertainty of the reconstruction and despite this having little impact compared to ML methods, it has also been shown that ML can bias the reconstruction to overrepresentation of common amino acids at each site (Williams et al., 2006; Hobbs et al., 2012). This can lead to less accurate but more stable and active enzymes, reason why ML methods can be preferred if ASR is used with protein engineering purposes (Spence et al., 2021).

The accessibility of ASR methods has increased in the last years with the implementation of algorithms as software and the implementation of automated pipelines on online servers, as summarized in **Table 1**. In fact, many methods used to perform phylogenetic analyses allow ancestral sequence reconstruction. Among these methods, PAML (latest version 4.7) stands out as one of the most used within ASR (Yang, 2007). The implementation of ASR methods on servers can be automated even to include as input only the unaligned sequences of interest, but the user is encouraged to revise reliability of the results and accuracy of the reconstructed sequences.

**Table 1:** Relevant methods to perform ASR.

Name	Description	url	Reference
PAML	Phylogenetic analysis of sequences using ML. CODEML or ML functions within PAML are the most popular tools to perform ASR based on ML.	http://abacus.gene.ucl.ac.uk/software/paml.html	(Yang, 2007)
MEGA11	Large and user-friendly collection of methods and tools of computational molecular evolution, including reconstruction of ancestral sequences based on ML or MP methods.	https://www.megasoftware.net	(Tamura et al., 2021)
BEAST2	Bayesian phylogenetic analysis with emphasis on time-scaled trees. Includes ASR.	https://www.beast2.org	(Bouckaert et al., 2019)
MrBayes	Program for the Bayesian phylogenetic inference allowing a wide range of models. Includes reconstruction of ancestral states.	https://nbisweden.github.io/MrBayes/index.html	(Ronquist et al., 2012)
FastML	Dedicated server for the reconstruction of ancestral sequences based on ML. It includes reconstruction of insertions and deletions, treated as binary data.	http://fastml.tau.ac.il	(Ashkenazy et al., 2012)
FireProt-ASR	Fully automated ancestral sequence reconstruction server. Indels are graphically represented in the results.	https://loschmidt.chemi.muni.cz/fireprotasr	(Musil et al., 2021)
GRASP	Automated server based on likelihood methods and graphical representation of ancestral sequences to treat insertion and deletions.	http://grasp.scmb.uq.edu.au	(Ross et al., 2022)
Ancescon	Distance-based phylogenetic inference and reconstruction of protein sequences. It considers the observed variation of evolutionary rates between positions to improve accuracy of the reconstruction.	http://prodata.swmed.edu/ancescon/ancescon.php	(Cai et al., 2004)
ProtASR2	ML based ASR accounting for structural information by using structural constrained substitution models	https://github.com/miguelarenas/protasr	(Arenas and Bastolla, 2020)

Most of the methods rely on empirical amino acid substitution models and assume that an entire sequence evolves at the same rate. ProtASR2 is an exception, as it allows inclusion of structural information to perform ASR (Arenas et al., 2017; Arenas and Bastolla, 2020). This method is based on substitution models that consider stability of the native, unfolded and misfolded states of a protein to avoid high and low hydrophobicity predictions, reconstructing proteins less biased towards higher stabilities (such as the ML methods based on empirical substitution models) and closer to the folding stability of simulated proteins (Arenas et al., 2015). Another important caveat when performing ASR is that the methods employed assume one unique phylogeny and that recombination events did not happen through evolution, despite recombination being a common and widespread genetic event. Pointing this out and addressing the issue, Arenas and Posada (2010a) analyzed the effect of recombination events in ASR studies. They showed that independently of the ASR method used, not considering recombination can bias ASR performed with nucleotides, codons and proteins, even at low simulated recombination events.

Finally, there is a handful of databases useful to collect the sequence information needed for ASR. Public databases constantly updated include those from NCBI (https://www.ncbi.nlm.nih.gov/) and UniProt (https://www.uniprot.org/), and in the case of biomass degradation two more specialized are JGI-Mycocosm (https://mycocosm.jgi.doe.gov/mycocosm/home) and CAZy (http://www.cazy.org/). There is also one recent database called Revenant, with hand curated information of resurrected proteins, that is linked to many other databases (https://revenant.bioinformatica.org/).

## 2 Fungal evolution

Given the high diversity of fungi, their evolution is sometimes difficult to study. There is a strong consensus that fungi (and nucleariids) are the sister group of holozoans within the clade Ophistokonta (Baldauf, 2008), and their divergence is usually estimated in the Mesoproterozoic/early Neoproterozoic era (~1000 mya) (Heckman et al., 2001; Parfrey et al., 2011). The last common ancestor of fungi is considered to have been non-filamentous and aquatic, with flagellated spores (James et al., 2006). However, some studies point to an earlier and different origin of fungi that would imply a revision of this phylogeny (Butterfield, 2005; Bengtson et al., 2017). The work by Bengtson and coworkers is especially controversial given the fact that they found fungus-like filamentous fossils originated in the early Paleoproterozoic era (~2400 mya), an origin considerably older (and different) than that previously thought. Additionally, it has been demonstrated that complex multicellularity in fungi appeared in different taxa as a convergent evolutionary adaptation, so the roots of these organisms have to be further investigated in detail (Nagy et al., 2018).

**Figure 2:**
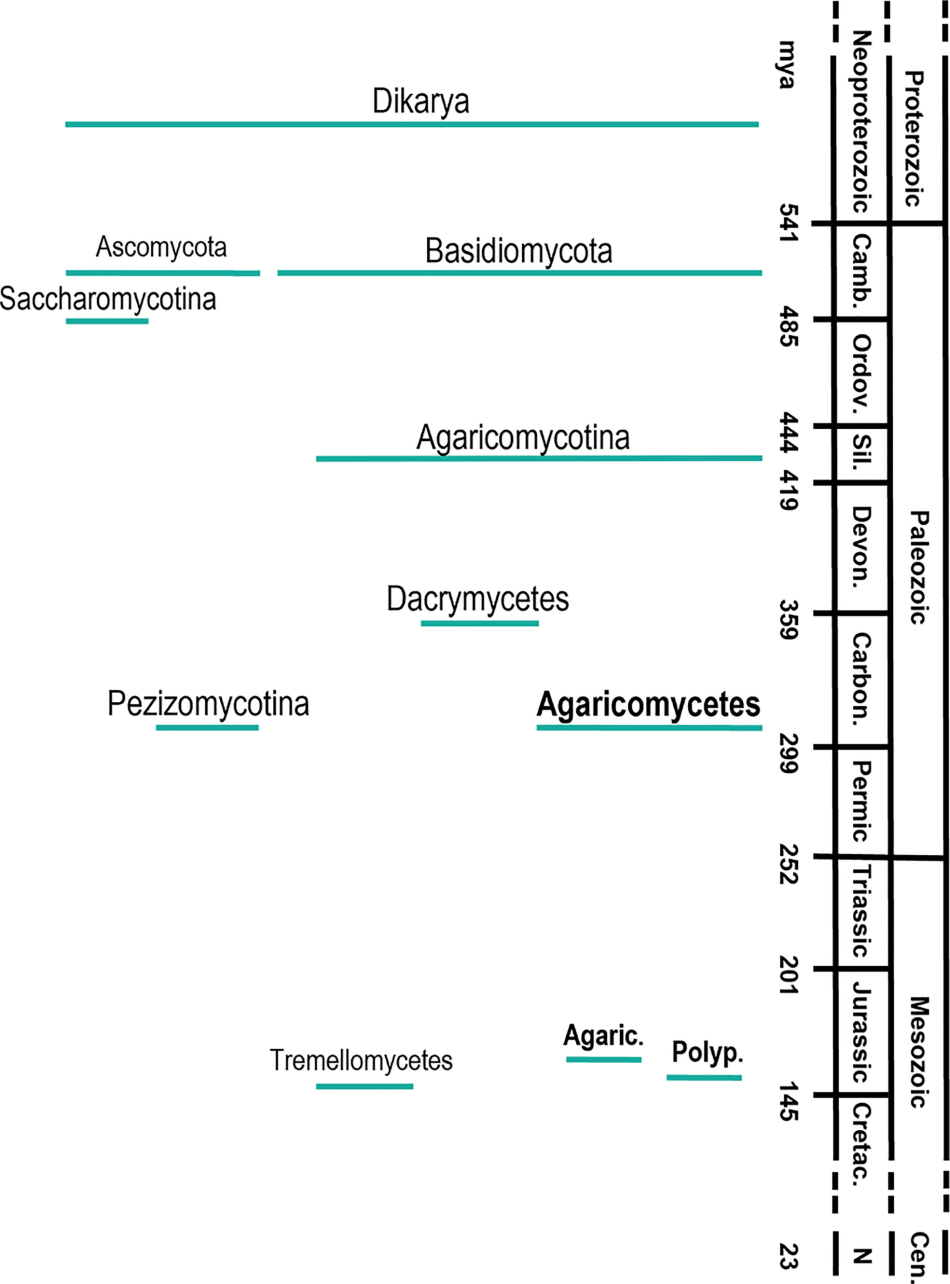
Timeline of the main diversification events in the evolution of wood-decay fungi in the subkingdom Dikaria, phyla Ascomycota and Basidiomycota, subphyla Saccharomycotina, Agaricomycotyna and Pezizomycotina, classes Dacrymycetes, Agaricomycetes and Tremellomycetes and orders Agaricales and Polyporales; Camb., Cambric period; Ordov., Ordovician; Sil., Sillurian; Devon., Devonian; Carbon., Carboniferous; Cretac., Cretaceous; Cen., Cenozoic era; N, Neogene period. Agaric, Agaricales; Polyp, Polyporales. The diversification times were taken from Floudas et al. (2012).

The origin and further evolution of Dikarya (including Ascomycota and Basidiomycota) is less troublesome (**Figure 2**). The time-calibration of different phylogenetic trees using fungal fossils establishes the origin of Dikarya in the Neoproterozoic era (~750 mya), and the divergence of Ascomycota and Basidiomycota in the Cambric period (~500 mya) (Parfrey et al., 2011; Floudas et al., 2012). The evolution of Basidiomycota shows a rapid and continuous diversification of the different groups that form the phylum (Zhao et al., 2017). Within them, the appearance of the class Agaricomycetes, where wood-rotting fungi are included, is estimated to occur at the end of the Carboniferous period (~300 mya) (Floudas et al., 2012; Zhao et al., 2017) with global geological consequences. The work of Floudas and coworkers postulates that the appearance of wood-rotting fungi, together with their production of the first lignin degrading enzymes, contributed to the end of biomass accumulation in form of coal at the end of the Carboniferous period. However, geoclimatic factors would have also significantly contributed to coal formation under ever-wet tropical conditions, and its decline could also be related to climatic shifts toward drier conditions (Hibbett et al., 2016; Nelsen et al., 2016).

Either way, after Agaricomycetes appeared there was a huge diversification of these fungi, generating the great number of species existing today (~21000). During this speciation, there was a complex evolution in terms of gene duplication/loss events, especially concerning PCWDE genes. In this sense, white-rot fungi (the most efficient lignin degraders) evolved with a higher ratio of gene duplication related to lignin-degrading enzymes, while brown-rot (with a preferential degradation of cellulose and limited degradation of lignin) suffered a clear loss of these genes (Ruiz-Dueñas et al., 2013; Nagy et al., 2017). Although the work of Floudas and coworkers (2012) suggested the common ancestor of Agaricomycetes as a white-rot fungus, a recent work involving the genome sequences of undersampled species shows that white-rot fungi evolved later in the Agaricomycetes (Nagy et al., 2016). Thus, the ancestor of both white- and brown-rot fungi could have been a poor wood degrader ("soft-rot") fungus, with an earlier diversification of wood carbohydrate active enzymes compared to ligninolytic peroxidases (although sampling more fungal genomes is necessary to clarify this point) (Nagy et al., 2016). It is important to note that the classic dichotomy white/brown-rot is useful to compare fungi, but it might be not representative of all degradation strategies. Aside from typical soft-rot decay, there are poor wood-degrading fungi displaying a white-rot like phenotype without ligninolytic genes, which could be a transition between the two classic phenotypes (Riley et al., 2014; Schilling et al., 2020).

### 2.1 Evolution of agaricomycetes and their PCWDE

Given the importance of lignin removal in the biorefinery context (Ragauskas et al., 2014), many efforts in the past decade were dedicated to sequencing Agaricomycetes genomes and to analyzing their lignocellulolytic enzyme machinery, together with the evaluation of the phylogenetic relationships of species and enzyme families involved in plant biomass degradation.

Especially relevant with respect to the evolution of wood-rotting fungi was the work of Floudas and colleagues (2012), who performed the first comprehensive study of Agaricomycetes genomes by analyzing the enzyme content through evolution, and the special role of ligninolytic peroxidases that will be discussed in detail below. Similar studies focused on Polyporales highlighted the differences between brown-rot and white-rot fungi in evolution, suggesting that igninolytic and related genes were lost in the brown-rot lineage (Ruiz-Dueñas et al., 2013; Ferreira et al., 2015). While the loss of ligninolytic heme peroxidases and the reduction of laccases seem clear in the brown-rot lineage, the enzymes involved in H_2_O_2_ production such as glucose–methanol–choline (GMC) oxidoreductases and copper-radical oxidases are widely distributed in the two lineages.

Recent works focused on the order Agaricales demonstrated that the expansion of ligninolytic genes is important not only for wood white-rot fungi, but also for fungi with other lignocellulose-decaying lifestyles (Ruiz-Dueñas et al., 2021). Moreover, one of the peroxidases of *Agrocybe pediades*, an example of grass litter fungus, displays lignin-degrading capabilities matching those of the white-rot ligninolytic enzymes (Sánchez-Ruiz et al., 2021), and one of the laccases of the same fungus, secreted during first days of solid-state fermentation of wheat straw, shows similar kinetics with lignin-derived phenols as those shown by laccases from white-rot Polyporales (Aza et al., 2021). Given that efficient lignin-decay capabilities appear in different nutritional modes in fungi with a varied PCWDE portfolio, the intriguing evolutionary relationships between distant taxonomical orders are still subject of intense investigation.

### 2.2 PCWDE families involved in lignocellulose degradation

**Figure 3:**
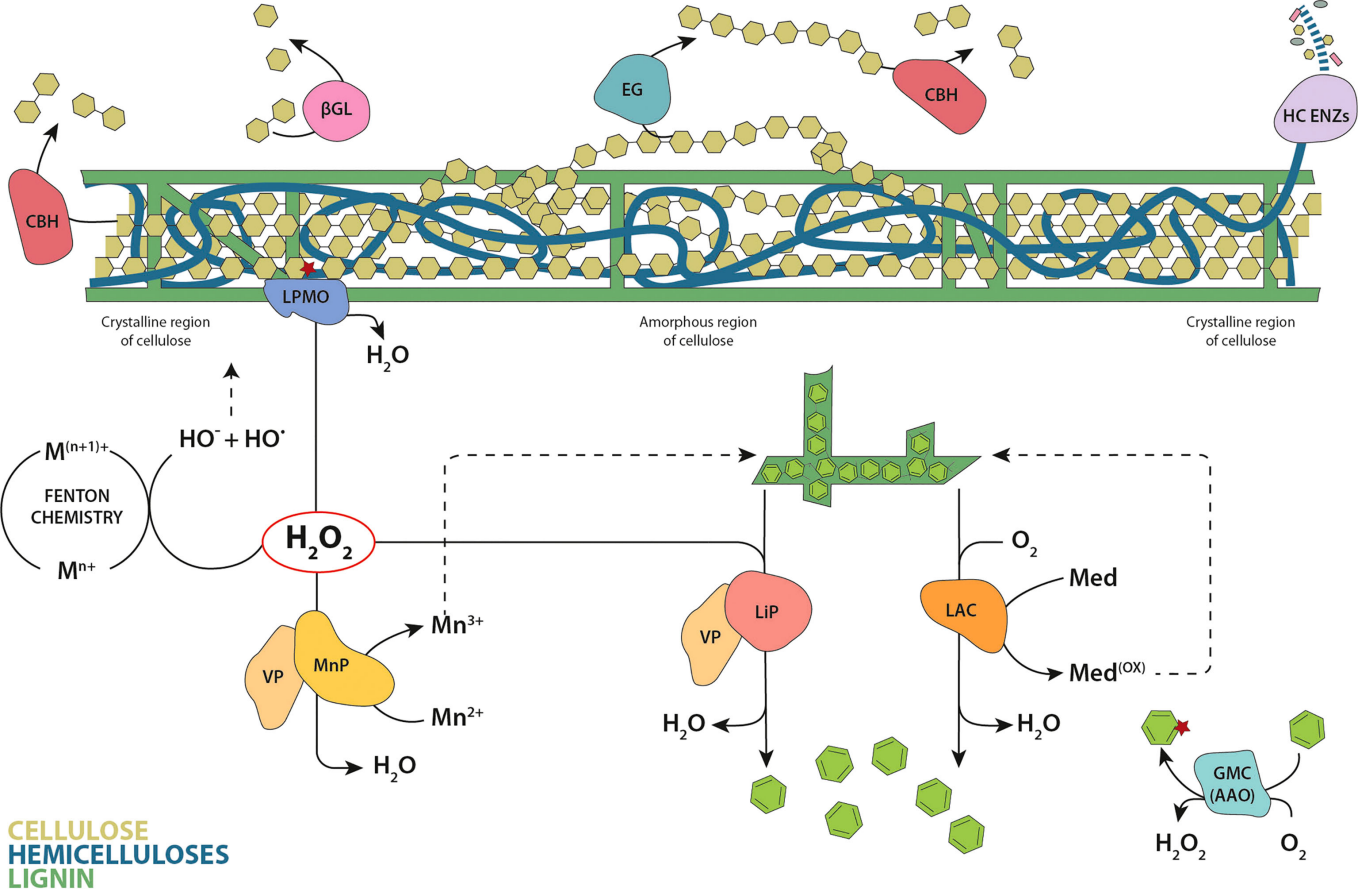
Classic model of enzymatic plant cell wall degradation. Hydrolysis of cellulose involves cellobiohydrolases (CBH) that hydrolyze the cellulose chains from reducing and non-reducing ends, endoglucanases (EG) that cut randomly inside the chains, and β-glucosidases (βGL) that convert the cellobiose units produced by CBHs to glucose. The oxidation of the crystalline cellulose surface by LPMOs results in an internal chain cleavage. The wide set of enzymes involved in the degradation of hemicellulose are represented by a single designation (HC ENZs). Laccases (LAC) directly oxidize the phenolic lignin moiety, or the nonphenolic lignin *via* redox mediators. Within peroxidases, lignin peroxidases (LiP) are able to oxidize directly the major nonphenolic lignin moiety, manganese peroxidases (MnP) oxidize Mn^2+^ to Mn^3+^ (the diffusible Mn^3+^ chelates oxidize the phenolic moiety of lignin) and versatile peroxidases (VP) catalyze the reactions described for both LiPs and MnPs. Brown-rot fungi also degrade cellulose and hemicellulose *via in vivo* Fenton chemistry. The glucose-methanol-choline oxidase/dehydrogenase superfamily (GMC) are exemplified by the aryl-alcohol oxidases (AAO) that generate hydrogen peroxide in the redox cycling of aromatic metabolites. Cellulose chains are depicted as linked olive-green hexagons, hemicelluloses in blue and lignin in dark green. Released lignin units and aromatic metabolites are represented as green hexagons.

Understanding the evolution of wood-rotting fungi requires the investigation of the enzyme families involved in lignocellulose degradation. Despite ligninolytic peroxidases having been extensively studied in the past decades due to their role in lignin degradation, wood-decay fungi secrete a consortium of different types of PCWDE. A rough classification can be made according to the main plant-cell wall component they act on. Here we will briefly describe the main families involved in biomass decay based on their role in lignin or cellulose/hemicellulose degradation (**Figure 3**), and we will outline the evolution of PCWDE in terms of ASR and enzyme resurrection works published to date. Given ASR is a relatively new technique, there is a limited number of publications so far, and in some cases, we extend the scope to related enzyme families from bacteria to show how ASR could help to better understand the evolution PCWDE. The works analyzed here, together with the methods for ASR employed by the respective authors, are summarized in **Table 2**.

**Table 2:** Summary of PCWDE works using ASR, and the methods used by the authors.

Protein family	Studied number of sequences	MSA building method	Phylogenetic tree reconstruction method	ASR method	References
Ligninolytic peroxidases^a^	113	MUSCLE	RAxML	PAML	(Ayuso-Fernández et al., 2018)
Ligninolytic peroxidases^a^	336	MUSCLE	RAxML	PAML	(Ruiz-Dueñas et al., 2021)
DyPs^b^	641	MAFFT	PhyML	PAML	(Zitare et al., 2021)
Laccases^c^	89	MUSCLE	MrBayes	PAML	(Gomez-Fernandez et al., 2020)
Endoglucanases^c^	32	MUSCLE	BEAST	PAML	(Barruetabeña et al., 2019)
Versatile lipases^b^	127	MUSCLE	ML(MEGA)	PAML	(Barriuso and Martínez, 2017)
Bacterial lipases^c^	83	MSAProbs	RAxML	PAML	(Rozi et al., 2022)

^a^Amino acid sequences used for ASR included as supporting information. ^b^Sequences retrieved from public databases, but not included as supporting information. ^c^Accession numbers of the sequences included as supporting information.

#### 2.2.1 Enzymes involved in lignin degradation

Lignin, the most abundant aromatic polymer in nature, is formed by phenyl-propanoid units derived from the oxidative radical coupling of cinnamyl alcohols catalyzed by peroxidases and laccases in the plant cell wall (Vanholme et al., 2010). It gives rigidity to plants while protecting cellulose against pathogen attacks. During plant evolution, its functions have been also linked to UV protection and water-proofing vessels (Weng and Chapple, 2010). The main fungal enzyme families related to lignin depolymerization are heme peroxidases and laccases, the former including class-II ligninolytic peroxidases (PODs) and to some extent the so-called dye-decolorizing peroxidases (DyPs). The GMC superfamily of oxidoreductases should be also considered here given their complementary role, even if they do not modify lignin directly.

*Ligninolytic peroxidases* are class-II heme peroxidases from the peroxidase-catalase superfamily (Zámocký et al., 2015), classified as AA2 in CAZy database (Lombard et al., 2014) and also known as PODs. They use H_2_O_2_ as the electron acceptor to start the catalytic cycle, and they can be classified into three families based on their catalytic properties: i) lignin peroxidases (LiP), enzymes able to oxidize directly the major nonphenolic moiety of lignin (Mester et al., 2001; Sáez-Jiménez et al., 2016); ii) short and long manganese peroxidases (MnP), performing the Mn^2+^ to Mn^3+^ oxidation, allowing the formation of diffusible Mn^3+^ chelates that oxidize the minor phenolic lignin moiety (Fernández-Fueyo et al., 2014); and iii) versatile peroxidases (VP), combining the catalytic properties of LiP and MnP (Ruiz-Dueñas et al., 2009). Fungal ligninolytic peroxidases have a crucial role in lignin degradation and carbon recycling in nature.

**Figure 4:**
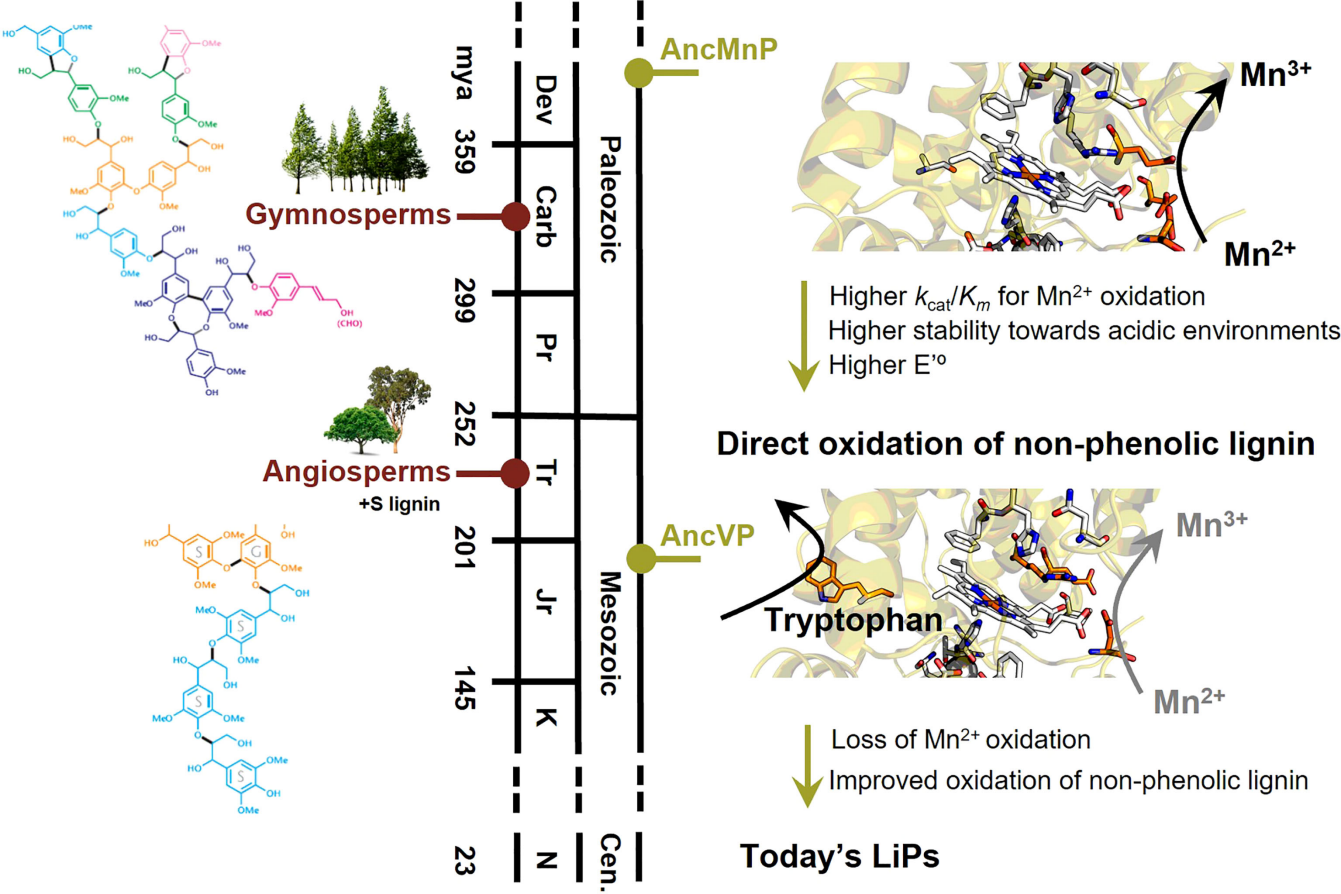
Main events in the evolution of ligninolytic peroxidases (right), in parallel with lignin evolution in gymnosperms and angiosperms (left). Initially, Mn^2+^ to Mn^3+^ oxidation was the main fungal strategy to oxidize lignin from gymnosperms, with only G units. This oxidation would be performed in the three acidic residues forming the Mn-binding site in ancestral MnPs (AncMnP). Once the solvent-exposed tryptophan appeared in the first ancestral VPs (AncVP), the direct oxidation of the major non-phenolic moiety of lignin would be possible and improved through the evolution of peroxidases up to today’s LiPs. Concomitant with the change in oxidation sites, the redox potential and the stability towards acidic pHs increased. In parallel, lignin became more complex and less phenolic with the appearance of angiosperms, including S lignin in its structure, and the above changes in ligninolytic enzymes were a fungal response to this increased complexity. The time-calibration was taken from (Ayuso-Fernández et al., 2019b) and (Morris et al., 2018).

The first ancestral character reconstruction studies on fungal PCWDE were done with these enzymes, showing that ancestral MnPs appeared in the Carboniferous period (around 400 mya) providing ancient fungi with new enzymatic tools to degrade recalcitrant polymers, and therefore contributing to the end of biomass accumulation in the form of coal (Floudas et al., 2012). According to the reconstruction of only specific amino acid positions involved in their catalysis, MnPs would have incorporated a solvent exposed catalytic tryptophan midway in the evolution of wood-rotting fungi, generating VPs. Later, ancestral VPs would lose the Mn-binding site, leading to the most efficient LiPs observed today (Floudas et al., 2012; Ruiz-Dueñas et al., 2013). This evolutionary hypothesis involving consecutive changes in the oxidation sites of ligninolytic peroxidases was experimentally proven by Ayuso-Fernández et al. (2017) performing ASR (and ancestor resurrection) in Polyporales, where most wood white-rot fungi are included. The authors sampled 10 Polyporales genomes obtaining a total of 113 curated ligninolytic peroxidase protein sequences to find key enzymes in the evolutionary trajectory towards the most efficient LiPs existing today. Their structural models showed the changes in oxidation sites proposed by Floudas et al. (2012), but ASR allowed the resurrection and characterization of the ancestral peroxidases in the laboratory to confirm the putative activities inferred *in silico*. The activity assays performed by the authors showed that the common ancestor of Polyporales peroxidases was a MnP, able to oxidize Mn^2+^ and low redox potential phenols, implying that ancestral Polyporales were in fact able to oxidize lignin, mainly using diffusible Mn^3+^-chelates. Ancestral MnPs diversified later into the types of ligninolytic peroxidases observed today, and the lineage leading to LiPs included an exposed catalytic tryptophan, generating ancestral VPs. These ancestral versatile enzymes would later lose the Mn-binding site, originating ancestral LiPs and the LiPs existing today, with only the surface exposed tryptophan as oxidation site. The authors showed that there was a progressive increase in the efficiency of Mn^2+^ oxidation until the incorporation of the surface tryptophan in VPs, indicating an initial preference for the cation diffusion strategy to degrade lignin. However, when the tryptophan appeared in evolution, direct oxidation of the major non-phenolic lignin moiety was possible, and that activity was improved (selected) in the evolution towards the most efficient LiPs, concomitant with the loss of their capability to oxidize Mn^2+^. This type of evolution incorporating a solvent exposed catalytic tryptophan happened several times and is a convergent trait in the evolution of white-rot fungi, as indicated by the convergence to a catalytic tryptophan independently in two different peroxidase lineages (LiP and VP) in Polyporales (Ayuso-Fernández et al., 2018). Direct oxidation of the major non-phenolic lignin was improved in both lineages, leading to extant LiPs or VPs, but through different changes in evolution and with different catalytic properties. Also, when the catalytic tryptophan appeared, enzyme stability in acidic pH, which is relevant to lignin oxidation in nature, was improved in both lineages. Together with the progressive changes in oxidation sites related to oxidative capabilities, the heme site was also reshaped through evolution. Using protein NMR and redox-potential measurement (**Figure 4**, right), the authors showed that the redox potential of ligninolytic peroxidases increased with time and was correlated with a subtle rearrangement in the coordination between the so-called proximal histidine and the iron of the heme cofactor (Ayuso-Fernández et al., 2019a).

The evolution of Polyporales peroxidases has also been related to plant evolution using lignins from different natural origins (Ayuso-Fernández et al., 2019b). Taking advantage of stopped-flow spectrophotometry and 2D-NMR, it was shown that ancestral ligninolytic peroxidases oxidized lignin from Gymnosperms better than lignin from Angiosperms while, after the incorporation of the solvent-exposed catalytic tryptophan, the preference changed to lignin from Angiosperms. Angiosperm lignin, whose appearance and diversification is more recent than Gymnosperm lignin (Morris et al., 2018), is more complex and less phenolic because it incorporates syringil (S) units, in addition to the guaiacyl (G) units synthesized by Gymnosperms. A time-calibrated peroxidase phylogeny indicates that the solvent-exposed tryptophan, which allows oxidization of the major non-phenolic lignin moiety directly, appeared near the time of Angiosperms diversification (**Figure 4**, left), pointing to co-evolution between a better defense against some pathogens, and other physiological/anatomical advantages in plants, and a better attack by more efficient lignin degradation in fungi (Morris et al., 2018; Ayuso-Fernández et al., 2019b).

More recently, peroxidase evolution was addressed in a wider array of ligninolytic fungi. 336 class-II peroxidases were identified in 42 genomes of Agaricomycetes, including not only Polyporales but also species of Agaricales, Russulales, Boletales and Amylocorticiales (Ruiz-Dueñas et al., 2021). ASR conducted with these enzymes corroborated the reconstruction studies described above for the Polyporales enzymes. In addition, two independent evolutionary pathways were identified leading to the appearance of the surface catalytic tryptophan in VPs and LiPs from Agaricales, with different transition enzymes compared to those reconstructed in Polyporales. Moreover, additional evolutionary pathways explaining the emergence of different novel MnP subfamilies in Agaricales and Russulales were identified. Although none of these novel MnPs or their reconstructed ancestors have yet been characterized, it has been suggested that their evolution in Agaricales most likely responds to an evolutionary adaptation to the different ligninocellulosic substrates (wood, decayed wood, grass litter or forest litter) on which these fungi grow in nature.

*Laccases* (EC 1.10.3.2, *p*-diphenol:dioxygen oxidoreductases, AA1_1 in CAZy), are multicopper oxidases (MCOs) that require O_2_ as electron acceptor and can directly oxidize a wide arrange of phenolic substrates, as well as aryl amines, N-heterocycles and benzenethiols (Xu, 1996; Baldrian, 2006). During their catalytic cycle, O_2_ is reduced to H_2_O as byproduct of the reaction. Given their low catalytic requirements, laccases have a great biotechnological potential. In fact, fungal laccases are the oxidoreductases with the highest number of applications reported to date in a range of industrial sectors, organic chemistry, pulp and paper, food and textile industries or detoxification of pollutants (Rodríguez Couto and Toca Herrera, 2006).

In fungi, MCOs play diverse physiological roles in morphogenesis, spore resistance and pigment formation, stress defense, fungal pathogen/host plant interaction, humus turnover and, as described previously, lignin degradation (Giardina et al., 2010; Janusz et al., 2020). In contrast to ligninolytic peroxidases, that can directly oxidize non-phenolic lignin, laccases have a relatively low redox potential that in principle restricts their oxidation action to the minor phenolic lignin moiety (around 20% of lignin polymer). However, this limitation is overcome in the presence of low molecular-weight compounds such as lignin-derived phenols that act as redox mediators of laccases (Camarero et al., 2005). Once oxidized by laccase, the mediator radical can oxidize recalcitrant substrates such as the nonphenolic subunits of lignin (Cañas and Camarero, 2010; Hilgers et al., 2018). Moreover, in the presence of lignin phenols laccase may contribute to fungal lipid peroxidation *in vivo*, thus expanding its role in the biodegradation of lignin and other recalcitrant aromatic compounds (Srebotnik and Boisson, 2005; Camarero et al., 2008) acting synergistically with MnPs.

Like ligninolytic peroxidases, laccases are found in high-copy numbers in white-rot fungi. An analysis of laccases *sensu stricto* in Polyporales suggested that there was only a single gene in the common ancestor of these fungi that appeared in the end of the Jurassic (Savinova et al., 2019). The authors hypothesized that the expansion of laccase genes happened in the second half of the Cretaceous era, relating it to the rise of a lignin more resistant to degradation with the expansion of Angiosperms, and in turn a need to increase the tools that fungi had to degrade plant biomass. By contrast, the results of a recent work covering diverse species of different orders from Agaricomycetes including Polyporales (Ruiz-Dueñas et al., 2021) showed that their ancient common ancestor (also dated in the late Jurassic) already possessed several *sensu-stricto* laccase genes from which the current laccases diversified.

The combination of ASR and directed evolution methods were shown to be an effective approach for lacasse engineering. The available evolutionary information helps to push the boundaries of enzyme design and promote the development of biocatalysts more suitable for industrial applications. In this way, ASR generates new starting points for enzyme design that are broadly different from the protein sequences of the extant enzymes. The resurrected enzymes can be excellent starting points for directed evolution to rescue promiscuous activities lost during natural evolution that can be used today to confer new enzyme functionalities for biotechnological purposes (Alcalde, 2015). A case in point was carried out in an ASR study using a final set of 87 laccase sequences from Basidiomycota fungi where three ancestral nodes were reconstructed (although only two of them could be biochemically characterized) (Gomez-Fernandez et al., 2020). As a proof of concept, the authors performed a preliminary directed evolution experiment using LacAnc100 ancestor, their most recent reconstructed sequence, towards the oxidation of β-diketones. The final laccase variant produced oxidation rates for 1,3-cyclopentanedione, the model compound used in the high-throughput screening assay, that were 160% higher than those of parental LacAnc100. In addition, the authors analyzed the S224G substitution in the OB-1 laccase obtained by directed evolution of PM1 basidiomycete laccase, which notably improved the activity detected for OB-1 compared to the native enzyme (Maté et al., 2010). This mutation was also an ancestral mutation shared by all resurrected ancestors. Therefore, the knowledge obtained by ASR can also be implemented in the engineering of contemporary enzymes by the incorporation of ancestral mutations.

*Dye-decolorizing peroxidases* are heme-containing peroxidases present in fungi, bacteria and archaea. They do not belong to the peroxidase-catalase superfamily, having a clear and distinct phylogenetic origin within the chlorite-dismutase superfamily (Zámocký et al., 2015). Fungal DyPs share a structural fold, and since their heme site and catalytic cycle are similar to the above ligninolytic peroxidases, a convergent evolution has been proposed (Linde et al., 2015). Their role in nature remains essentially unknown, and as peroxidases they are overall inefficient, indicating that this might not be their physiological role (Singh and Eltis, 2015), which correlates with their little impact in Agaricales lifestyle evolution (Ruiz-Dueñas et al., 2021). However, related to lignin oxidation, it seems that some fungal DyPs can modify lignin (Linde et al., 2021), but there is an ongoing debate whether other types of DyPs are able to truly oxidize this recalcitrant polymer given their poor performance or the amounts of enzyme needed to observe any effect (Min et al., 2015; Rai et al., 2021). Traditionally, DyPs have been classified in classes A, B, C and D according to primary structural homologies, but a more recent classification considering tertiary structure was proposed, resulting in the alternative designations P, I and V. The details of DyP classification are beyond the scope of this review, but for clarity, the majority of fungal DyPs were classified in family D belonging to the recent type V.

There is only one work using ASR to study fungal DyPs (Zitare et al., 2021), where the authors obtained an ancestral D-type enzyme with the main purpose of establishing a good system to characterize DyPs better. They used 641 sequences from Basidiomycota fungi and reconstructed two ancestral nodes from their phylogeny. Unfortunately, only one of those ancestors (together with an alternative variant to cover ambiguity in the reconstruction) was expressed as a soluble protein. The full characterization of their ancestral DyP showed that its capability to oxidize typical DyP substrates is diminished. Intriguingly, the ancestral DyP is able to oxidize Mn^2+^, despite apparently having no Mn^2+^-binding site, which could be linked to an ancestral role in lignin degradation. However, since time calibration was not performed, the correlation of this activity with the ancestral MnPs discussed above is difficult. Interestingly, the authors used their ancestral setup to perform structure-function mutagenesis studies focusing on conserved amino acids in the distal heme site. Their results showed the first direct evidence of the role of two conserved residues in the heme site, related to H_2_O_2_ reduction during the catalytic cycle of DyPs. This work shows how ancestral enzymes are useful not only to evaluate evolutionary hypotheses, but also to characterize unknown structural features of enzyme families.

*GMC oxidoreductases*, forming the glucose-methanol-choline oxidase/dehydrogenase superfamily, assist in the degradation of lignin (and crystalline carbohydrates) by generating the H_2_O_2_ used by peroxidases (and lytic polysaccharide monooxygenases, LPMOs), and also by reducing oxidized lignin products to avoid repolymerization (Marzullo et al., 1995). It is a varied group including aryl-alcohol oxidase (AAO), alcohol (methanol) oxidase, cellobiose dehydrogenase, glucose oxidase, glucose dehydrogenase, pyranose oxidase and pyranose dehydrogenase, and all together form the AA3 family in CAZy (Sützl et al., 2019). This large and diverse family of enzymes share a common structural fold that possesses a flavin binding motif for the adenine dinucleotide (FAD) cofactor. There are no ASR works related to fungal GMC oxidoreductases, but ancestors from mammalian flavin-containing monooxygenases (FMOs), proteins that also hold a FAD-binding motif, were resurrected by ASR (Nicoll et al., 2020). These FMO ancestors possessed a well-conserved FAD binding domain and were active, pointing out that ancestral proteins containing this co-factor can be obtained and making future reconstruction of ancestral GMC oxidoreductases plausible.

#### 2.2.2 Enzymes involved in cellulose and hemicellulose degradation

Cellulose is the main component of the plant cell wall and the most abundant polymer on Earth, being an essential renewable source in the biorefinery context (Payne et al., 2015). Despite its simple chemical composition (β-1,4 linked anhydroglucose units), the degradation of cellulose in nature requires the concerted action of multiple enzymes, including both hydrolases and oxidoreductases. The three classical types of glycoside hydrolases (GH) (outlined in detail below) act synergistically on amorphous regions of the cellulose fibers (**Figure 3**). However, the action of lytic polysaccharide monooxygenases (LPMOs) is required to break cellulose crystallinity, boosting with it the action of GHs. In addition, brown-rot fungi also degrade cellulose and hemicellulose *via in vivo* Fenton chemistry (**Figure 3**).

*Endoglucanases* (EG, endo-1,4-β-D-glucanases, EC 3.2.1.4) randomly cleave β-1,4 bonds in internal amorphous regions of cellulose, generating new non-reducing ends. Their fold can be varied, but all of them display a large cleft with the catalytic amino acids in order to accommodate cellulose fibrils (Davies and Henrissat, 1995). In CAZy, they are classified in structural families GH5, GH6, GH7, GH9, GH12, GH44, GH45, and GH74 (Couturier et al., 2016).

*Cellobiohydrolases* (CBH, cellulose 1,4-β-cellobiosidases, EC 3.2.1.91) release cellobiose (the glucose disaccharide) from cellulose fragments released by EGs. They can act on reducing or non-reducing ends (Couturier et al., 2016), and are processive enzymes meaning that they can slide through the cellulose fiber to continue cleavage. They are included in families GH6 and GH7 in CAZy.

*β-glucosidases* (BGL, EC 3.2.1.21) cleave the cellobiose dimer into glucose monomers. They are not processive enzymes, and with the cleavage of cellobiose they cause product inhibition on CBHs, a bottleneck in cellulose degradation (Sørensen et al., 2013). In CAZy they are included in families GH1 to GH3.

There are no examples of fungal ancestral GHs, but a reconstruction of ancestral EGs was carried out using 32 sequences of the EG Cel5A family from bacteria (Barruetabeña et al., 2019). Since the carbohydrate-binding modules (CBM) of the sequences are very heterogeneous and poorly conserved, only the catalytic region was considered for the ASR. The results indicate that the LFCA_EG ancestor, belonging to the oldest node of Firmicutes phyla, is a promising biocatalyst for biotechnological applications since it displays high activity over a broad range of temperature and pH values, and it acts on different substrates and displays high heterologous expression yields. Its thermostability and good activity at high temperatures are important features for commercial applications in the pulp and paper industry or the conversion of cellulosic biomass into fermentable sugars for biofuels production (Bayer et al., 2004; Patel et al., 2019). Interestingly, LFCA_EG also shows processive endoglucanase and exoglucanase activity, although no sequences coding hydrolases with this activity were included in the phylogeny. Unlike the fungal cellulases, the bacterial cellulases can be involved in assembling multi-enzyme complexes, called cellulosomes, together with other enzymes related to the degradation of lignocellulose (Bayer et al., 2004). In this sense, LFCA_EG enhances its activity when it is assembled into a minicellulosome.

As an example of direct biotechnological application, the LFCA_EG ancestor was tested in the transformation of cellulosic resources into nanocellulose (nano-sized form of cellulose), which is a high value material suitable for high-performance applications such as tissue engineering. Surprisingly, this enzyme can single-handedly produce pure nanocellulose particles bellow 500 nm with elevated stability, crystallinity, and controlled aspect ratio. This was reported to be the first attempt to generate nanocellulose by a single enzyme in an efficient manner (Alonso-Lerma et al., 2020).

*Lytic polysaccharide monooxygenases (LPMOs)* are mono-copper enzymes with a characteristic flat surface capable of breaking the crystallinity of cellulose (and chitin) by hydroxylation of the 1 or 4 position of the β-1,4 glycosidic bond. Their discovery is recent (Vaaje-Kolstad et al., 2010), but since LPMO discovery many types with different properties and roles in nature have been described, forming families AA9-AA11 and AA13-AA17 in CAZy. Fungal LPMOs belong classically to family AA9, but some AA13 and AA16 fungal LPMOs have been described. The high diversity of AA9s (even within the same species) has been related to their distinct action on cellulose and complex and varied hemicelluloses (Monika et al., 2022). Together with the recent discovery showing their use of H_2_O_2_ as the true co-substrate in nature (Bissaro et al., 2017), and their strong connections in co-expression matrix analyses (Arntzen et al., 2020), LPMOs became a central piece in enzymatic biomass degradation. A first study on LPMO ASR has been recently reported, showing how the reshape of their surfaces could be important for substrate specificity (Ayuso-Fernández et al., 2022).

*Hemicelluloses degradation.* Hemicelluloses are the third major component of plant cell walls, after cellulose and lignin. They possess a complex chemical structure, being a mixture of branched polysaccharides with three types of backbones and many types of substitutions (Saha, 2003; van den Brink and de Vries, 2011). Degradation of hemicelluloses requires a myriad of enzymes, both for the cleavage of the backbones and the release of the substitutions in those backbones. This multienzymatic and complex degradation is out of the scope of this work, but has been reviewed in detail elsewhere (van den Brink and de Vries, 2011; Li et al., 2022).

*Carbohydrate-binding modules*. All the aforementioned enzymes acting on cellulose or hemicellulose frequently contain an additional domain or domains that guide the binding to the polymer, known as carbohydrate-binding modules (CBM) (Boraston et al., 2004). CBMs are usually small in size, flexible and stable, and they are widely distributed in nature (Liu et al., 2022). There is a massive number of CBMs annotated in CAZy, currently classified in 91 families. The evolution of these noncatalytic domains is complex, with many such domains appearing across different enzyme families, which could indicate domain transferences between genes and species.

### 2.3 Lipases

Lipolytic enzymes (EC 3.1.1) are ubiquitously produced in nature and they encompass a diverse group of hydrolases catalyzing the cleavage and formation of ester bonds (Ali et al., 2012). Their classification is complex, and several systems have been used over time (Sarmah et al., 2018). Although they do not directly modify cellulose/hemicellulose or lignin, their main role in biomass degradation has been related to the synergistic effect of feruloyl esterases with cellulases, xylanases and pectinases to degrade complex carbohydrates (Wong, 2006; Arntzen et al., 2020). Fungal lipases also interact with cuticle, the cutin polyesters and waxes barrier that covers plants aerial surfaces (Arya et al., 2021).

Roughly, lipases catalyze the hydrolysis or synthesis of a broad range of different carboxylic esters, showing high specificity towards glyceridic substrates. Sterol esterases (EC 3.1.1.13) show the same capability, but acting on sterol esters as their natural substrates (Hasan et al., 2006; Vaquero et al., 2016). However, in fungi it has been reported that a group of enzymes included in the *Candida rugosa* (recently designated *Diutina rugosa*) like lipase family (abH03.01, homologous family in the Lipase Engineering Database) show a broad substrate specificity combining both lipases and sterol esterases properties acting on acylglycerols and sterol esters. Due to the wide specificity of members of this group, it has been proposed to reclassify them as “Versatile Lipases” (Barriuso et al., 2016). These enzymes have been related to the initial degradation of epicuticular waxes and cuticle (Heredia, 2003). Degradation of these external layers makes the plant polysaccharides more accessible to the different CAZymes.

Structurally, all enzymes mentioned above possess a substrate-binding pocket consisting of a large hydrophobic cavity covered by a mobile amphipathic α-helix (named lid or flap). The lid stays closed in an aqueous solution under physiological conditions. However, when the enzyme is in the presence of hydrophobic substrates, the lid rearranges its position (creating an open gate) and the catalytic site becomes accessible (Rodríguez-Salarichs et al., 2021).

To study the evolution of the versatile lipases in fungi, 127 sequences from Agaricales were selected for ASR (Barriuso and Martínez, 2017). Unfortunately, in this study the predicted ancestral proteins were not expressed, so experimental characterization was not available, and conclusions had to be drawn from in silico predictions related to amino acid sequences and 3D homology models. The inferred sequences corresponding to ancestral nodes selected as key intermediates in the evolution showed that both the number of amino acids and hydrophobicity increase throughout the versatile lipases’ lineage in the lid region. In fact, homology models from intermediate nodes showed that the lid of the oldest ancestors held a loop instead of the α-helix present in the lid of extant lipases. Thus, the gradual extension of this region might have led to the formation of the α-helix. It is important to note that the lid is responsible for substrate recognition, so changes in the hydrophobicity and length of this region may be involved in substrate specificity (Mancheño et al., 2003). Similarly, the size of the putative substrate-binding pocket as well as the length of the intramolecular tunnel also changed through evolution (Barriuso and Martínez, 2017). The substrate-binding site is located in an internal and hydrophobic tunnel, whose shape and amino acid composition could be related to the different substrate specificities and catalytic properties of these enzymes (Mancheño et al., 2003). A hypothesis based on similar enzymes is that a longer intramolecular tunnel might enable the communication of the substrate-binding pocket with the outside of the protein, creating a putative novel exit of the reaction products.

Although not directly related to the scope of this review, we switch kingdoms to focus on lipolytic enzymes from bacteria to discuss a couple examples of ancestral lipases characterized experimentally. Bacterial lipases are divided into an increasing number of families with the identification and characterization of novel enzymes. In particular, family I (true lipases) is the largest family and is divided into several subfamilies (Arpigny and Jaeger, 1999; Jaeger and Eggert, 2002; Kovacic et al., 2018). An ASR study was carried out using 83 sequences of bacterial lipases, and the oldest node was selected for resurrection (Rozi et al., 2022). Family 1.3 was selected for ASR due to their singularity in structure, amino acid sequences and secretion mechanism. The last common ancestor of family 1.3, which the authors named “LUCA”, showed remarkable properties related to its substrate profiling with a wider promiscuity for medium and long chain substrates (C10-C16 substrates) compared to present-day 1.3 lipases. It was hypothesized that evolution could have directed modern 1.3 lipases to prefer medium chain substrates. On the other hand, LUCA displayed an optimum temperature at 70°C, which is one of the highest optimum temperatures observed for other modern 1.3 lipases. The LUCA ancestral enzyme possessed other biochemical characteristics such as thermostability, operability at high pH values, and tolerance towards different organic solvents, making it a potentially valuable biocatalyst for industry. Again, this result suggests that the optimum temperature of modern 1.3 lipases decreased through evolution to adapt to environmental conditions. An extreme example of this evolutionary adaptation would be the cold-adapted lipase AMS8 isolated from an Antarctic *Pseudomonas* that reached the optimum activity at 30°C (Ganasen et al., 2016).

## 3 Applicability of ASR in protein engineering

Biomass pretreatment and enzymatic hydrolysis are the most expensive steps in biomass upcycling in biorefineries (Payne et al., 2015). For that reason, significant efforts have been made to not only discover new and more efficient enzymes, but also to improve the existing enzymes through protein engineering (Percival Zhang et al., 2006; Alcalde, 2015). ASR becomes then an excellent tool to explore an increased sequence space with non-existing proteins, mining for enzymes with interesting catalytic properties.

Aside from its use in developing evolutionary hypotheses, ASR potential in protein engineering is well documented (Risso et al., 2018; Spence et al., 2021). One of the typical features usually observed in ancestral enzymes is their higher thermostability, with improved melting temperatures of up to +30°C compared with extant enzymes (Trudeau et al., 2016). Commonly, this feature is noticed in proteins predicted for up to a billion years ago (Risso et al., 2014), but it is not perfectly clear if this is an artifact of reconstructions based in ML methods or a true trait of ancestral proteins (Wheeler et al., 2016). A good example of the ASR methods employed to obtain stable variants is the production of a hyper-thermostable L-amino acid oxidase used to perform deracemization to D-amino acids. With a Tm > 95°C and long-term thermal stability, this enzyme is a perfect candidate for industrial application (Ishida et al., 2021).

ASR is used also to study structure/function relationships. Sampling intermediates in evolution can give essential clues to understand how active sites are shaped, which is crucial to engineer new activities or properties in enzymes (Voordeckers et al., 2012; Ayuso-Fernández et al., 2018; Spence et al., 2021). Also, ASR can aid in better understanding of enzyme function to guide protein engineering efforts. In this case ASR methods have been used to show unprecedent allosteric mechanisms (Gamiz-Arco et al., 2021), and to study the evolution of protein-protein interactions (Jemth et al., 2018). Ancestral enzymes are useful also to expand the reactions observed in nature with artificial enzymes. Catalytic promiscuity through evolution is an ongoing debate (Copley, 2015; Glasner et al., 2020), but if the family of interest displays an array of activities, ASR can be used to obtain versatile enzymes or even catalysts with unique reactivities (Huang et al., 2012; Voordeckers et al., 2012; Babkova et al., 2017).

## 4 Conclusions

Since Pauling and Zuckerkandl conceived the idea of paleogenetics, the development of ASR methods has led to a continuously increasing number of ancestral proteins resurrected in the laboratory, both for protein engineering purposes and to explain evolutionary histories. This is the case also for the limited number of ASR works studying wood-rotting fungi. But this must be seen as an opportunity: ASR has only scratched the surface of possibilities in these fascinating organisms. Most of PCWDE families are still not studied with ASR, and in those already studied, there is still room to resurrect more ancestors or to analyze different subfamilies and study them in the laboratory. Here we have reviewed the current knowledge of ancestral enzymes of wood-rotting fungi, together with a summary of the methods available to do ASR. We foresee a growing number of works in the upcoming years consolidating ASR as an important tool to study fungal evolution and to improve the applicability of fungal enzymes.

## Author contributions

IA-F and GM reviewed the literature and wrote the initial draft. FR-D, SC, and ATM performed the critical revision of the article. All the authors participated in the final editing and approved the submitted version. 

## Funding

This research was funded by the Genobioref (BIO2017-86559-R) and Lig2Plast (PID2021-126384OB-I00) projects of the Spanish Ministry of Science and Innovation (co-financed by FEDER funds); by the WoodZymes (H2020-BBI-JTI-2017-792070) and CuBE – ERC Synergy Project (H2020-ERC-2019-SyG-856446) EU projects, and by the Consejo Superior de Investigaciones Científicas project PIE-202120E019 and grant 2021AEP106, SusPlast platform, and program for the Spanish Recovery, Transformation and Resilience Plan funded by the Recovery and Resilience Facility of the European Union, established by the Regulation (EU) 2020/2094. G.M. acknowledges The Tatiana Pérez de Guzmán el Bueno Foundation for his predoctoral Environment grant. Finally, we acknowledge support of the publication fee by the CSIC Open Access Publication Support Initiative through its Unit of Information Resources for Research (URICI).

## Acknowledgments

We thank the editor and the referees for their time and effort spent to improve our review.

## Conflict of interest

The authors declare that the research was conducted in the absence of any commercial or financial relationships that could be construed as a potential conflict of interest.

## Publisher’s note

All claims expressed in this article are solely those of the authors and do not necessarily represent those of their affiliated organizations, or those of the publisher, the editors and the reviewers. Any product that may be evaluated in this article, or claim that may be made by its manufacturer, is not guaranteed or endorsed by the publisher.
